# Parasite Evolution and Life History Theory

**DOI:** 10.1371/journal.pbio.1000524

**Published:** 2010-10-19

**Authors:** Beth F. Kochin, James J. Bull, Rustom Antia

**Affiliations:** 1 Department of Biology, Emory University, Atlanta, Georgia, United States of America; 2 Section of Integrative Biology and Institute for Cellular and Molecular Biology, University of Texas, Austin, Texas United States of America; 3 Department of Biology, Emory University, Atlanta, Georgia, United States of America

As a group, parasites are extraordinarily diverse. Even closely related parasites may behave very differently, infecting different host species, causing different pathologies, or infecting different tissues. For example, *Escherichia coli* bacteria, a typically harmless inhabitant of the human gut, can, in different forms, cause diarrhea, intestinal bleeding, urinary tract infections, kidney bleeding, meningitis, and other diseases [1]. Underlying this diversity is evolution.

It is widely appreciated that parasites are prone to rapid evolution, and because of their often short generation times and large population sizes, parasites may evolve far more rapidly than their hosts. Attempts to understand parasite evolution, and the relevance of that evolution to disease, go back at least half a century to the first observations of drug resistance evolution in bacteria [2]. However, the application of evolutionary theory to parasites remains fertile ground for original research [3]. Indeed, evolutionary biology and parasitology have undergone such rapid advances in recent years that it has been difficult to keep abreast of both. Some recent papers, including the study of Babayan et al. in this issue of *PloS Biology* [4], apply results from one branch of evolutionary theory—life history theory—to the characteristics of pathogens of medical interest such as parasitic roundworms (nematodes) and malaria [5]. Babayan et al. propose that the life history of parasitic microfilarial worms shows evidence of adaptive “plasticity.” Specifically, they propose that worm development inside a mammalian host changes in response to the host's immunity, and that the parasite's response matches predictions from life history theory.

Most basically, life history theory addresses the birth and death schedule of an organism in the context of its environment: how is natural selection expected to shape an organism's age of first reproduction, its fecundity, and survival? (See [6]–[9] for reviews.) Body size and other phenotypic traits are also often considered in the theory. As a typical example, a population that loses half its individuals each year to predation is expected to evolve to begin reproducing at a younger age than a population losing only 10% of its individuals to predation annually. This occurs even though early reproduction has costs that would reduce lifetime fecundity if predation is low. This early maturity increases the chance that that an individual survives to maturity, a feature that is increasingly important with an increasing mortality rate. As might be expected intuitively, increases in juvenile mortality select for earlier maturation, while increases in adult mortality do not have this effect [10],[11]. The specific life history that will evolve by natural selection depends on details such as the different mortality rates and fecundity schedules that accrue to individuals with different ages of maturation.

In order to avoid telling just-so stories about the evolution of life history traits, it is important to make predictions and then test them. It is hard to make predictions for a single species, in a single environment, at one point in time. The difficulty is that we do not know what life history options are available to the organism, and unless those options are known, prediction is hard. To escape this dilemma, life history theory applications have developed almost completely in a comparative context, predicting how birth and death schedules should vary across populations of the same species in different environments. If population P1 inhabits environment E1 and population P2 inhabits environment E2, the theory leads to straightforward relative predictions based on the differences between E1 and E2. These predictions are easy because the birth–death constraints of the organism will be the same for population P1 as for P2, so even though we cannot predict the exact birth–death schedules favored in E1 and E2, we can predict the direction of the differences (whether the population in E1 should mature earlier or produce more offspring early in life, for example).

The situation encountered in the study of Babayan et al. is slightly more complex. Their problem does not involve two separate populations of the same parasite in different environments, but instead involves one parasite population responding to different environments. It is of course ubiquitous for organisms to live in variable environments: the seeds of a single plant may encounter a range of soil moistures and sunlight availabilities to germinate; a parasite may encounter hosts having different levels of immunity. One possible response of the organism is to evolve a compromise life history, one that produces the best average response to all conditions. Another possibility is that the organism evolves a plastic response: in each environment the organism displays a different life history suited to that environment. Whether a fixed or plastic response is optimal depends on the costs of sensing and regulation versus the benefits of plasticity. An approach taken to determine if an organism displays adaptive phenotypic plasticity is to compare the responses in the two environments and see if the differences observed are consistent with what might be expected if the organism had optimized to the two environments.

## Phenotypic Plasticity in Nematodes

**Figure 1:**
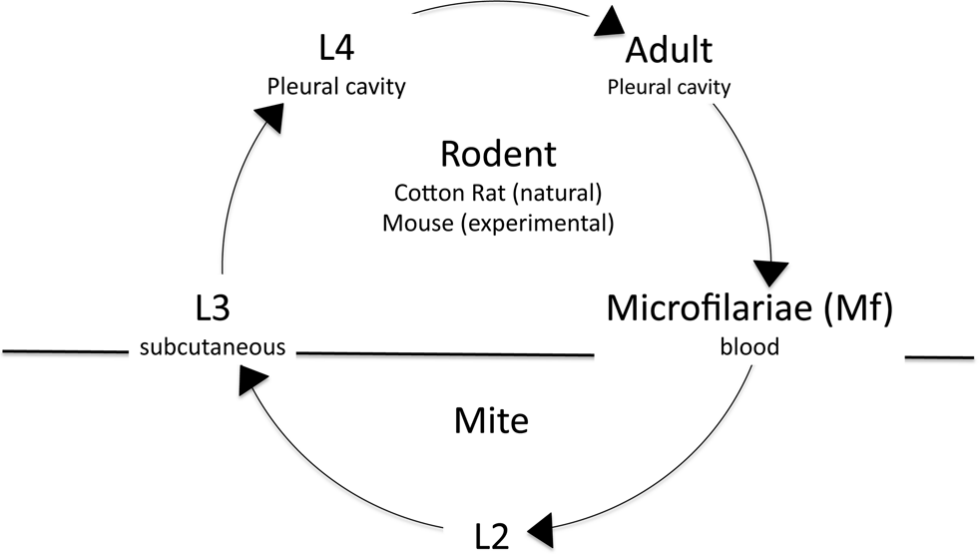
The life history of the filarial nematode. The L3 stage of the pathogen enters the vertebrate natural host (the cotton rat) by the bite of a mite where it matures via the L4 stage into adults that produce many tiny Mf (microfilariae) that are responsible for transmission. Mf are taken up by the arthropod vector and mature via the L2 stage to the L3 stage that is transmitted to the vertebrate host. The experimental system uses subcutaneous injection of L3 to to start the infection in inbred laboratory mice.

The organism studied by Babayan et al., *Litomosoides sigmodontis*, is a nematode used as a model of human filarial diseases. The complex life cycle of the parasite is shown in Figure 1. For the purpose of this discussion, infection of a rodent begins with inoculation of larvae by an arthropod vector. The larvae then mature to adults that produce the transmission (microfilariae, Mf) stage (additional stages in the vector can be ignored for the present). We focus on the endemic situation where fitness is proportional to the total transmission (*R_0_*) rather than the rate of transmission. We assume that the rate of transmission at a given time is proportional to the number of Mf present at that time; in this case the fitness of the parasite will be proportional to the area under the curve of a plot of the density of Mf over the course of infection.

Life history theory predicts that the optimal combination of rates of maturation from larvae to adult, of subsequent adult survival, and of the production of Mf, will result in maximizing transmission, i.e., maximization of the area under the curve of the transmission stage. Host immunity is important because immunity impairs worm survival, much like the presence of a predator would impair prey survival. The level of immunity of the host is a major source of variability for the within-host environment of the worm. Different levels of immunity can arise because of genetic differences in the host, as well as from differences in the infection history of the host.

**Figure 2:**
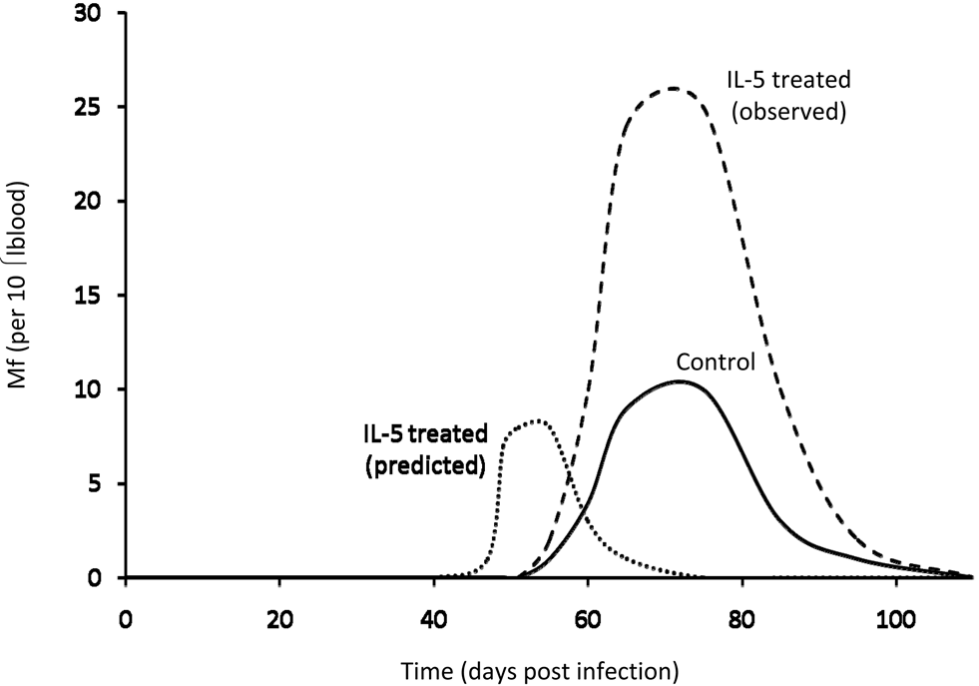
Predictions and observed results. A sketch of how the density of the transmission (Mf) stage is altered by IL-5 treatment. The adaptive phenotypic plasticity hypothesis predicts that in the presence of additional IL-5 the parasite should produce Mf earlier and/or at a more rapid rate, but the total amount of Mf produced should be less than in the controls. In contrast with the prediction of the adaptive phenotypic plasticity hypothesis, total Mf production was much greater in the IL-5–treated mice.

This brings us to the question addressed by Babayan et al.: how does the parasite respond to hosts with different levels of immunity to the early (pre-adult) parasite stages? As immunity acts by increasing parasite mortality, they predict that the parasite will show adaptive phenotypic plasticity by having earlier maturation and investment in the transmission stage in immune hosts, even though it would be at the expense of transmission later on in the infection. The earlier investment could be achieved by earlier generation of Mf or by an increased rate of Mf production. The cost of this earlier investment in transmission would be reflected in a smaller area under the curve of the Mf stage. The predictions are shown in Figure 2.

In order to test their hypothesis, Babayan et al. performed the following experiments. They first showed (using mice lacking the ability to make the IL-5–induced eosinophil response) that the IL-5–induced eosinophil response results in more rapid maturation of the larval L3 to the subsequent L4 stage, as might be expected from an adaptive phenotypic plasticity hypothesis (but unexpectedly they observed no impact on the generation of adults). The second experiment was designed to test the effect of early immune responses on worm fecundity. Two groups of mice, a control group and an immune group, were infected with nematode larvae. The immune group, for technical reasons, was not generated by immunization, but instead by injection of the cytokine IL-5 together with the L3 larvae. Injected IL-5 is associated with the recruitment of eosinophils directed against the early stages of the parasite (e.g., the L3 larvae). Surprisingly, they found that IL-5–treated mice produced more transmission stages Mf at all times in comparison with the controls (see Figure 2, and also Figure 4B of Babayan et al.). At face value, the increased total production of Mf in these mice rejects the adaptive phenotypic plasticity hypothesis, which predicts that control mice should produce as many or more Mf over the course of infection in comparison with IL-5–treated mice. As Babayan et al. point out, there are a number of ways to explain this surprising result.

One hypothesis is that the unexpected results for total transmission arise because of differences between the laboratory mice used in the study and the natural hosts of the parasite. In this case, the results should not be interpreted in an adaptive context.

The second hypothesis is that the worm does not exhibit adaptive phenotypic plasticity in response to IL-5. It is not known why parasite development is enhanced by IL-5. The fact that total Mf production is higher with IL-5 than without IL-5 could reflect the parasite having optimized to an environment with high immunity and using IL-5–induced eosinophilia as a signal for reproduction. The parasite in this case has not optimized to a naive or low IL-5 environment, or its reproductive output would be higher in the absence of immunity than in the presence of immunity. Furthermore, we see little support for the prediction of the adaptive phenotypic plasticity hypothesis regarding the change in timing of Mf production—the Mf curve in the presence of IL-5 looks essentially like a scaled version of the control instead of a curve shifted to earlier reproduction, as in our Figure 2.

A third hypothesis, and the one favored by Babayan et al., allows the adaptive phenotypic plasticity hypothesis to be retained by proposing that there must be a hidden cost elsewhere—for example, the Mf produced in IL-5–treated mice may be less fit than those produced in untreated mice. We favor the first two hypotheses over the third because they are consistent with the existing data without requiring the additional assumption of a hidden cost required by the third hypothesis.

Babayan et al. point out further experiments are needed to distinguish between these hypotheses. The first hypothesis could be tested by determining whether the outcome of the experiments described above differs when the parasite infects its natural host (the cotton rat) rather than laboratory mice. Transmission experiments (ideally using vectors to transmit the parasite between vertebrate hosts) could be used to discriminate between the second and third hypotheses. If there is less transmission from the IL-5–treated mice than the untreated mice (because the Mf in the IL-5–treated mice are less fit), then we would favor the third hypothesis.

Discriminating between these possibilities is not only an academic exercise. Which hypothesis is true may have important consequences for vaccination. If the parasite has optimized to an environment with high IL-5 (second hypothesis) then vaccination that increases IL-5 without providing much further immunity could result in increased transmission from immunized individuals following infection. If the adaptive phenotypic plasticity hypothesis (third hypothesis) is correct, then we don't expect this problem—vaccinated individuals will not transmit more than unvaccinated ones.

## Adaptive Phenotypic Plasticity in Malaria Infections

Life history theory has been applied to the allocation of resources between the growth and transmission stages during the blood stage of malaria infections [4],[12],[13]. The optimal allocation of resources between growth and transmission stages may change in environments that increase the mortality of the parasite, such as after the generation of immunity during the course of an infection, or after drug treatment. At high levels of mortality, when extinction of the parasite is certain, the parasite should invest mainly in the transmission stage. At low levels of mortality, the parasite should increase allocation into the growth stage as this prevents clearance of the parasite.

An interesting recent study by Reece et al. applied these ideas to drug treatment by proposing that the parasite should display adaptive phenotypic plasticity in response to the severity of the treatment [5]. At high drug doses the parasite should increase investment in the transmission stage, consistent with earlier in vitro observations [14],[15]. At low drug doses the parasite should decrease investment in the transmission stage, and they demonstrated this to be the case in an in vitro setting. At present, the test of adaptive plasticity is incomplete, and it will be exciting to see the outcome as the dose of a given drug is changed from low to high, as well as similar experiments in an in vivo system.

## Evolutionary and Adaptive Considerations

The application of evolutionary principles to infectious disease certainly holds much promise, but also offers many challenges [3],[16],[17]. As is the case in all of evolutionary biology, particular care should be taken before assuming an “adaptationist program” [18].

Evolutionary biologists would like to understand how a trait (such as a plastic life history response) has evolved, and the forces (selection, drift, etc.) driving this evolution. When selection is involved, we would further like to know the reason behind the selection. Because we don't have time machines allowing us to go back and observe and perform experiments on organisms in the past, we are forced to take indirect approaches. For example, to investigate whether an observed instance of phenotypic plasticity is adaptive, one typically considers evolutionary history or the current utility (optimality) of the plasticity.

An evolutionary history approach asks: is the plasticity evolving as expected by natural selection? This requires evidence that (i) the phenotypes of interest have evolved from the ancestral state and (ii) the evolution is in the direction expected (by natural selection on that trait).

An optimality approach asks: is the current plasticity beneficial? In other words, are the phenotypes of interest in different environments optimal in those environments (two for simplicity)? As it is difficult to determine the optimal response in any environment, the approach taken is to determine whether the phenotypic differences in the two environments are in the direction predicted by optimality considerations.

Both the Babayan et al. and Reece et al. papers take the optimality approach, as it is the most accessible and the approach we must often accept when evolutionary principles are first applied to a problem.

It is worth noting that the answer from one approach does not necessarily tell you the answer from the other [19]. Current optimality does not imply the trait has evolved for that reason (noses might not have been selected to carry glasses [18]). Likewise, the reader can imagine a case where a recent change in the environment has caused a trait to be non-optimal, but selection is driving the trait towards optimality.

Finally, we note that understanding whether a trait is beneficial may have uses in addition to understanding its evolution. For example, as discussed earlier, whether filarial nematodes exhibit adaptive phenotypic plasticity in response to immune responses may have implications for vaccination.

## Conclusions

Evolutionary life history theory offers potential explanations for a number of perplexing parasitological puzzles with public health importance. Why do parasites use signals from the immune system to control their development [20]–[22]? Why does the malaria parasite change its rate of differentiation into the transmission stage following drug treatment [5],[15],[23]? While recent studies by Reece et al. [5] and Babayan et al. [4] are some of the first papers to use evolutionary life history theory to address these questions, we suggest that the acceptance of their adaptive explanations will require more evidence than has been presented so far.
